# The Potential Role of Genic-SSRs in Driving Ecological Adaptation Diversity in *Caragana* Plants

**DOI:** 10.3390/ijms25042084

**Published:** 2024-02-08

**Authors:** Qinglang Wang, Xing’er Chen, Yue Meng, Miaomiao Niu, Yuanyuan Jia, Lei Huang, Wenhong Ma, Cunzhu Liang, Zhiyong Li, Liqing Zhao, Zhenhua Dang

**Affiliations:** 1 Ministry of Education Key Laboratory of Ecology and Resource Use of the Mongolian Plateau & Inner Mongolia Key Laboratory of Grassland Ecology, School of Ecology and Environment, Inner Mongolia University, Hohhot 010021, China; qlwang0405@163.com (Q.W.); 18353906000@163.com (X.C.); 13836376701@163.com (Y.M.); nmiaom@163.com (M.N.); jyy773839714@163.com (Y.J.); huanglei_1996@163.com (L.H.); whma@imu.edu.cn (W.M.); bilcz@imu.edu.cn (C.L.); zylee007@imu.edu.cn (Z.L.); zhaotieniu@126.com (L.Z.); 2 Collaborative Innovation Center for Grassland Ecological Security, Ministry of Education of China, Inner Mongolia Autonomous Region, Hohhot 010021, China

**Keywords:** *Caragana*, genic-SSR, ecological adaptability diversity, functional trait, climate factor, transcriptome sequencing

## Abstract

*Caragana*, a xerophytic shrub genus widely distributed in northern China, exhibits distinctive geographical substitution patterns and ecological adaptation diversity. This study employed transcriptome sequencing technology to investigate 12 *Caragana* species, aiming to explore genic-SSR variations in the *Caragana* transcriptome and identify their role as a driving force for environmental adaptation within the genus. A total of 3666 polymorphic genic-SSRs were identified across different species. The impact of these variations on the expression of related genes was analyzed, revealing a significant linear correlation (*p* < 0.05) between the length variation of 264 polymorphic genic-SSRs and the expression of associated genes. Additionally, 2424 polymorphic genic-SSRs were located in differentially expressed genes among *Caragana* species. Through weighted gene co-expression network analysis, the expressions of these genes were correlated with 19 climatic factors and 16 plant functional traits in various habitats. This approach facilitated the identification of biological processes associated with habitat adaptations in the studied *Caragana* species. Fifty-five core genes related to functional traits and climatic factors were identified, including various transcription factors such as MYB, TCP, ARF, and structural proteins like HSP90, elongation factor TS, and HECT. The roles of these genes in the ecological adaptation diversity of *Caragana* were discussed. Our study identified specific genomic components and genes in *Caragana* plants responsive to heterogeneous habitats. The results contribute to advancements in the molecular understanding of their ecological adaptation, lay a foundation for the conservation and development of *Caragana* germplasm resources, and provide a scientific basis for plant adaptation to global climate change.

## 1. Introduction

Adaptation is a fundamental biological phenomenon that reflects the intricate relationship between organisms and their indispensable environment [1]. With global climate change, all life forms on Earth face significant challenges in maintaining their existence and reproductive success. Under these circumstances, a wide range of genetic variations has emerged at various scales [2,3,4]. These variations further undergo a rigorous culling process shaped by the forces of individual selection, population dynamics, species-level dynamics, and the overarching influence of the natural environment. At the end of this selection, advantageous variations are filtered and consolidated into heritable adaptive strategies, ensuring the perpetuation of traits enhancing survival fitness across generations. 

Microsatellites, also known as simple sequence repeats (SSRs), are genomic elements characterized by short motifs, typically 1–6 bases in length, that are tandemly repeated up to a few dozen times [5]. Widely distributed across the genomes of eukaryotes, these sequences are known for their co-dominant and nearly neutral inheritance, as well as their high variability [6,7]. Initially considered “junk” DNA devoid of biological function, our understanding of genome composition and function has evolved, revealing that these repetitive sequences play cryptic roles in driving genome structural variation, gene family diversity, and gene function variation. SSRs are found in both gene coding and regulatory regions, referred to as genic-SSRs, and they possess a greater capacity to increase gene instability compared to their genomic counterparts [8]. Numerous studies have demonstrated a correlation between the length variation of microsatellites and changes in gene expression. Specifically, longer microsatellite lengths are associated with lower gene expression under non-stress conditions; conversely, they exhibit higher expression under stress conditions [9]. In sunflower (*Helianthus annuus*), the allele length of microsatellites affects gene expression related to homeopathic and trans-regulatory functions [10]. In *Camellia sinensis*, some transcription factor families are rich in TF-SSR, which might contribute to the regulation of its abiotic stress response [11]. For example, GRAS, MYB, and WRKY are rich in AT repeats, while others, such as GATA, are rich in CG repeats. The (CA)_n_ repeats in the promoter region of the *Tilapia prll* gene, encoding a hormone involved in osmotic regulation, exhibit diversity that is linked to the expression and salt effects of the *prll* gene [12]. In the era of “omics”, identifying such genetic variation from non-model species that widely inhabit diverse natural environments will significantly enhance post-genomic studies, providing the potential to further unravel the theories and mechanisms governing organisms’ responses to climate change.

*Caragana* is a native desert perennial shrub belonging to the Leguminosae family, and is widely distributed in sandy grasslands and desert regions of west and northwest China and Mongolia [13]. There are more than 100 species in *Caragana* genus. The leaves of *Caragana* plants are mostly hardened with keratin or wax layers to reduce water evaporation, and the well-developed root system is utilized to absorb water and stabilize dry soil. Some of the *Caragana* species are known for their drought, cold, and heat resistance, and they have strong adaptability to windbreak and sand fixation in sandy environments [14]. Therefore, the *Caragana* species can serve as stress-tolerant or nominal urban plants to promote urban green infrastructure (UGI) adaptation to climate change [15] while improving the overall quality of urban environments for both human habitats and wildlife populations [16]. In addition, *Caragana* plants can fix atmospheric nitrogen, form protective forests for crops and pastures, provide supplementary livestock feed (leaves and flowers) and fuel energy (shoots) for local farmers, and have high ecological value [17]. 

It has been observed that *Caragana* plants exhibit distinctive distribution patterns, reflecting the evolution of species-specific characteristics in morphology, physiology, and biochemistry [18]. This makes them good candidates for conducting research on plant adaptability [17,19,20,21]. For instance, *C. versicolor* is distributed in southern Sichuan, Xizang, and Qinghai, while *C. polourensis* is observed on the northern slope of Kunlun Mountain in Xinjiang and the northern slope of Qilian Mountain in Gansu. *C. brachypoda* predominantly scatters across piedmont plains, low slopes, and stabilized sandy lands in the semi-desert regions of Inner Mongolia [22]. So far, extensive research has delved into understanding the adaptive strategies of *Caragana* plants, spanning various domains, including morphological anatomy [19,20,21], geographical distribution [22,23], physiological ecology [22,24,25,26,27], photosynthetic characteristics [22,24,25,26,27], phylogenetic analysis [26,27], and population genetics [28,29,30,31,32]. With the advent of advanced gene sequencing technologies, new frontiers have been opened in unraveling the molecular mechanisms governing stress resistance and adaptive evolution within the genus. Pioneering studies include the construction of a transcriptome database for *C. korshinskii* [33], and the transcriptomic exploration of seedlings’ responses to salt and drought stress in the species [34]. Zhu et al. [35] identified the expression characteristics of microRNAs under salt stress in *C. intermedia*, while Ning et al. [36] unveiled the regulatory role of genes downstream of the gibberellin and cytokinin signaling pathways, along with cell skeleton-related genes, in trichome development of *C. microphylla*, shedding light on their involvement in drought resistance. Wan et al. [13] illuminated the multifunctionality of the *C. intermedia WRKY* gene family, demonstrating that the overexpression of *CiWRKY75-1* and *CiWRKY40-4* impedes drought resistance and retards leaf senescence in *Arabidopsis*, providing a groundwork for further exploration of WRKY transcription factor-mediated drought resistance mechanisms. In a recent study [37], it was observed that under moderate and severe water deficits, *Caragana* leaves exhibited an accelerated generation of O_2_^•−^ and H_2_O_2_. These investigations have unveiled the outcomes of long-term natural selection and adaptation, illuminating the unique structural underpinnings and survival tactics that enable *Caragana* plants to thrive in arid environments amid environmental heterogeneity. However, most of these studies focus on one or a few species of *Caragana*, and there is a lack of macro-level investigations into the ecological adaptability of a broader spectrum of species.

In the present study, we focused on 12 species of the *Caragana* genus distributed in Inner Mongolia, Tibet, and Xinjiang, China. Taking into consideration the selective effects of heterogeneous environments on the adaptability of different species, we utilized transcriptome sequencing technology and related bioinformatics analysis to identify polymorphic genic-SSRs of various *Caragana* plants. Through the analysis of gene expression characteristics, the relationships between genic-SSR variation and related gene expression, plant functional traits and habitat climate factors in *Caragana* were identified, and the impact of SSR variations on specific gene functions, metabolic pathways, cellular processes was discussed. Our results enhance the understanding of the correlation between the genic-SSR variations and the adaptability fitness of different *Caragana* species to their habitats and provide novel insights into their ecological adaptation mechanisms.

## 2. Results

### 2.1. Sequencing Outputs and Assembly

A total of 40.53 GB of polymerase reads were obtained from a *C. korshinskii* sample using PacBio SMRT sequencing technology, with an average length of 75,219.18 bp and an N50 of 134,134 bp. Following data filtering, clustering, correction, and redundancy removal, 278,746 unique isoforms (uniq-isoforms) were obtained, with an average length of 981 bp, an N50 of 1569 bp, and a GC content of 41.11%. BUSCO analysis indicated that approximately 95% of the isoforms were full-length transcripts, including complete and single-copy as well as complete and duplicated transcripts. Among the isoforms, 244,991 were annotated in the non-redundant (NR) protein databases, the Nucleotide (NT) sequence databases, the Swiss-Prot databases, the euKaryotic Orthologous Groups (KOG) databases, the Kyoto Encyclopedia of Genes and Genomes (KEGG) databases, the Gene Ontology (GO) databases, and the Pfam databases, with the number of annotated genes in each database being 222,002 (79.64%), 233,378 (83.72%), 168,486 (60.44%), 167,028 (59.92%), 171,436 (61.50%), 182,009 (65.30%), and 135,165 (48.49%).

For short-read sequencing, a total of 177.29 GB of clean data were obtained from 38 *Caragana* cDNA libraries. The clean reads in all samples had a Q20 greater than 97%, and the Q30 ranged from 88.28% to 93.85%. Following de novo assembly, 47,146 to 112,247 unigenes were generated across the 38 datasets (Table S1).

### 2.2. Identification of Polymorphic Genic-SSRs

**Figure 1:**
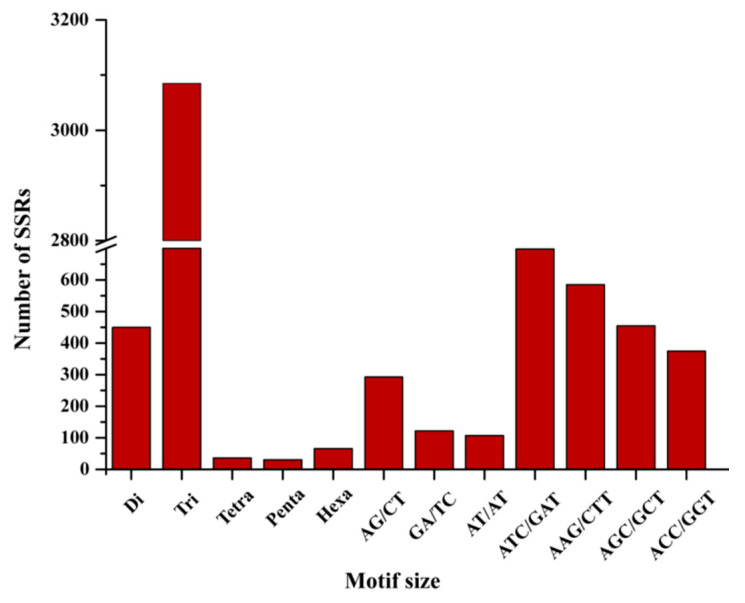
Identification and frequency analysis of polymorphic genic-SSRs. The *x*-axis represents the five SSR types along with the motif type exhibiting the highest frequency within each SSR class, while the *y*-axis denotes the number of identified genic-SSRs.

A total of 3666 polymorphic genic-SSRs were identified in 38 *Caragana* datasets, representing 111 motif types within 3545 isoforms. Trinucleotides were the most abundant motif type (3085, 84.15%), followed by dinucleotides (450, 12.27%). Quadnucleotides, pentanucleotides, and hexanucleotides were far less prevalent. Among the trinucleotide repeats, ATC/GAT (698, 22.63%) emerged as the dominant motif type, followed by AAG/CTT (585, 18.96%), AGC/GCT (454, 14.72%), and ACC/GGT (375, 12.16%). The most abundant dinucleotide was AG/CT (293, 65.11%), followed by AT/AT (107, 23.78%) and AC/GT (50, 11.11%) (Figure 1).

### 2.3. Location Prediction and Frequency Analysis of the Polymorphic Genic-SSRs

**Figure 2:**
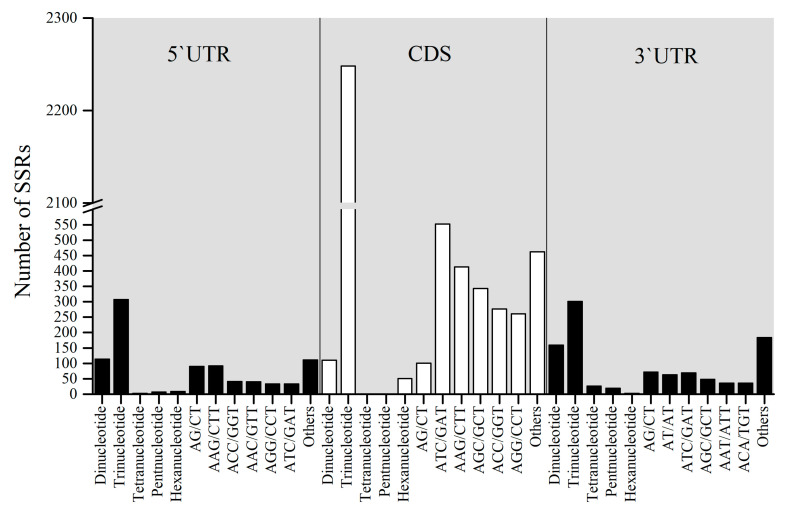
Distribution of polymorphic genic-SSRs within relevant isoforms. The *x*-axis represents the distribution of polymorphic genic-SSRs and motif sequence types, while the *y*-axis indicates the numbers of identified genic-SSRs.

In total, 20,774 genic-SSRs were located in the coding sequences (CDSs) and untranslated regions (UTRs) of 3545 isoforms sequence. Of these genic-SSRs, 440, 2409 and 508 genic-SSRs in 434, 2355, and 499 isoforms were distributed in 5′UTRs, CDSs, and 3′UTRs, respectively. Most of the trinucleotide repeats (93.32%) were located in CDSs, while most of the dinucleotide repeats (31.30%) were located in 3′UTRs. GAA/TTC, TCA/TGA, ATC/GAT, GCA/TGC, ACC/GGT, CAA/TTG, AAG/CTT, CAC/GTG, and CCA/TGG were relatively abundant in the CDSs. ATC/GAT (79, 7.9%) was the most abundant repeat in 5′UTRs, and GAA/TTC (106, 8.7%) was the most abundant repeat in 3′UTRs (Figure 2).

### 2.4. Functional Annotation of the Polymorphic SSR-Containing Sequences

GO functional annotation indicated that 2765 isoforms, containing 2683 genic-SSRs, align with 5578 GO terms. The three major GO category annotation results were as follows: 1521 (27.27%) for biological process, 1847 (33.11%) for cellular components, and 2210 (39.62%) for molecular function. Within each category, molecular function comprised 1314 GO terms. Isoforms with 5′UTR-SSRs and CDS-SSRs were primarily annotated to the “cellular process” term, accounting for 148 and 723 isoforms, respectively. 3′UTR-SSR-isoforms were predominantly enriched in the “metabolic process” term, with 128 isoform sequences. The cellular components included 303 GO terms, with the representative term being “cellular component”. Isoforms related to 5′UTR-SSRs, 3′UTR-SSRs, and CDS-SSRs, totaling 217, 225, and 1218, respectively, were associated with this term. Molecular function comprised 657 GO terms, with isoforms related to 5′UTR-SSRs (173), CDS-SSRs (964), and 3′UTR-SSRs (236) primarily enriched in the “binding” term (Figure S1).

Pathway analysis assigned 1300 isoforms containing genic-SSRs to 104 KEGG pathways. “Metabolic pathways” emerged as the most abundant pathway in 5′UTRs, CDSs, and 3′UTRs, followed by “Biosynthesis of secondary metabolites”, “Spliceosome”, and “RNA transport” (Figure S2).

### 2.5. WGCNA Analysis of Polymorphic Genic-SSRs Related Differentially Expressed Genes on Functional Traits

**Figure 3:**
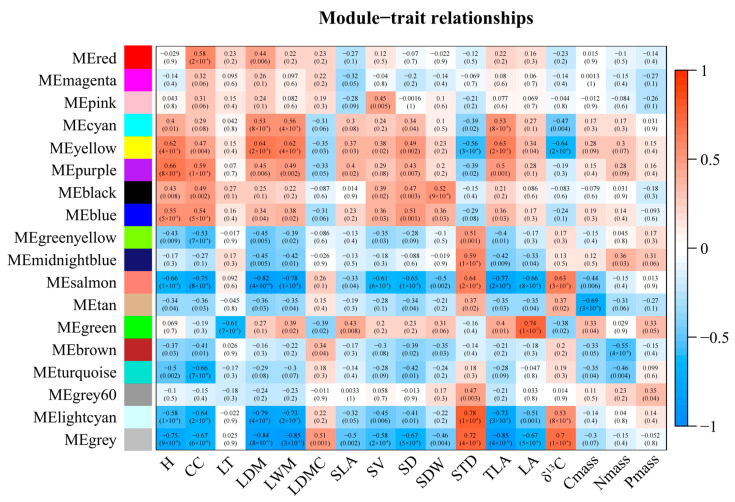
Correlation heatmap of co-expression gene modules and functional traits in *Caragana*. The horizontal axis represents various functional traits, while the vertical axis represents the eigenvectors of each module. Red and blue cells indicate positive and negative correlations, respectively.

After filtering, 2424 differentially expressed genes containing genic-SSRs were identified. GM_2 was identified as an outlier sample (Figure S3) and excluded from subsequent analyses. WGCNA analysis revealed that six modules (|r| ≥ 0.65, *p* < 0.05) exhibited significant correlations with the sixteen functional traits of *Caragana*. Among these modules, the purple and salmon modules were positively and negatively correlated with the trait of plant height (H), respectively. The salmon and turquoise modules were negatively correlated with crown coverage (CC), while both the salmon and turquoise modules showed negative correlations with leaf dry mass (LDM) and leaf wet mass (LWM). The salmon module also displayed a negative correlation with stem diameter (SD). The green module exhibited a positive correlation with leaf area (LA), whereas the salmon module showed a negative correlation with LA. The light cyan module was positively correlated with stem tissue density (STD). Both the salmon and light cyan modules were negatively correlated with total leaf area (TLA), and the tan modules exhibited a significant negative correlation with carbon biomass (Cmass) (Figure 3).

**Figure 4:**
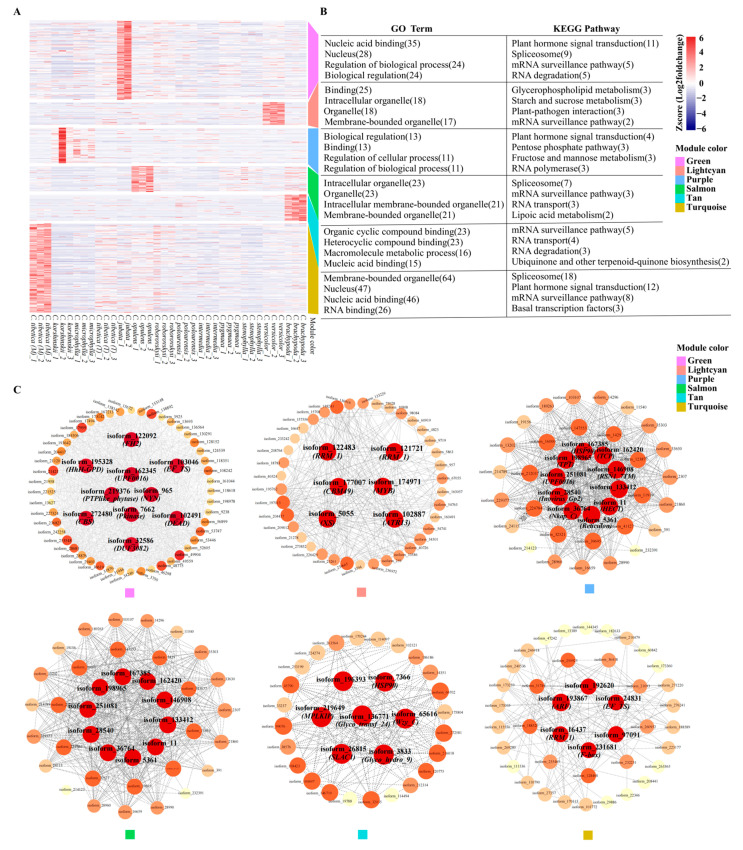
Clustering, enrichment analysis, and identification of hub genes in functional trait-specific modules containing differentially expressed genes associated with genic-SSRs. (**A**) The heatmap illustrates the expression profiles of genes within six specific modules (green, light cyan, purple, salmon, tan, and turquoise) across 38 *Caragana* samples. (**B**) Functional enrichment results for Gene Ontology (GO) and the Kyoto Encyclopedia of Genes and Genomes (KEGG) are presented, where each of the six segments delineates module functions in a top-to-bottom arrangement. (**C**) Hub genes within these six modules are highlighted, with the dot size and color intensity reflecting the level of connectivity—darker colors denote higher connectivity, signifying hub genes.

GO enrichment analysis revealed that differentially expressed genes (DEGs) within the six modules (green, light cyan, purple, salmon, tan, and turquoise) were primarily enriched in GO terms associated with “membrane-bounded organelles”, “nucleus”, “RNA binding”, “binding”, and “biological regulation”, etc. Additionally, KEGG enrichment analysis identified predominant enrichment of these genes in pathways such as “plant hormone signal transduction”, “spliceosome”, and “mRNA surveillance pathway” (Figure 4B).

Among the six modules, ten, six, ten, six, seven, and six hub genes were identified, respectively (Figure 4C). Pfam domain annotation revealed that *FH2*, *EF_TS*, *CBS*, etc., constituted the main gene group in the green module. Hub genes in the light cyan module included *MYB*, *CBM49*, *RRM_1*, and *XS* gene families. The purple module was characterized by hub genes mainly belonging to *HECT*, *Hsp90*, *Reticulon*, etc. In the salmon module, hub genes included *Fasciclin*, *RRM_1*, *Hsp90*, etc., while the tan module contained hub genes such as the *Glyco_transf_24*, *MPLKIP*, and *Glyco_hydro_9* gene families. *RRM_1*, *EF_TS*, and *F-box* were identified as the primary hub genes in the turquoise module (Table S2). 

### 2.6. WGCNA Analysis of Polymorphic Genic-SSRs Related Differentially Expressed Genes on Climate Factors

**Figure 5:**
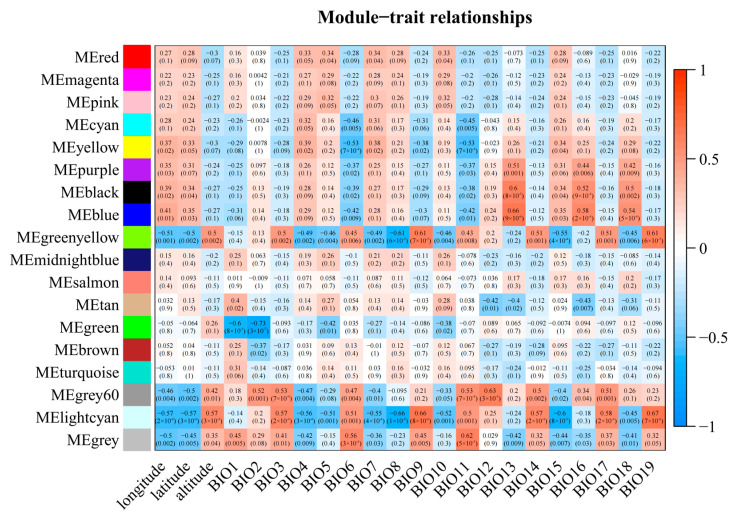
Heatmap depicting the correlation between co-expression gene modules and climate factors in *Caragana* sampling locations. The horizontal axis represents various climate factors, while the vertical axis represents the eigenvectors of each module. Red cells indicate positive correlation, while blue cells represent negative correlation.

Three modules were found to be significantly correlated with the climatic factors of *Caragana* sample locations (|r| ≥ 0.65, *p* < 0.05). The green and light cyan modules were negatively correlated with mean diurnal range(BIO2) and mean temperature of the wettest quarter (BIO8), respectively. The light cyan module was positively correlated with mean temperature of the driest quarter (BIO9) and precipitation of the coldest quarter (BIO19), while the blue module was positively correlated with precipitation of the wettest month (BIO13) (Figure 5).

**Figure 6:**
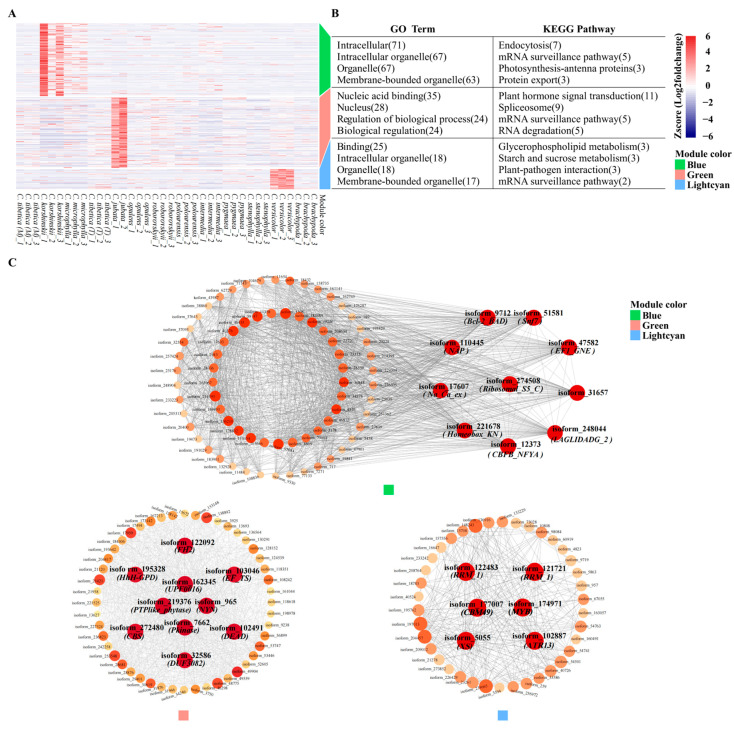
Clustering, enrichment analysis, and identification of hub genes in climate factor-specific modules containing differentially expressed genes associated with genic-SSRs. (**A**) The heatmap illustrates the expression profiles of genes within three specific modules (blue, green, and light cyan) across 38 *Caragana* samples. (**B**) Functional enrichment results for Gene Ontology (GO) and the Kyoto Encyclopedia of Genes and Genomes (KEGG) are presented, where each of the three segments delineates module functions in a top-to-bottom arrangement. (**C**) Hub genes within these three modules are highlighted, with the dot size and color intensity reflecting the level of connectivity—darker colors denote higher connectivity, signifying hub genes.

GO enrichment analysis revealed that DEGs in the three modules (blue, green, and light cyan) were primarily enriched in GO terms related to “intracellular organelle” “biological regulation”, “intracellular”, “binding”, etc. KEGG enrichment analysis identified that these genes were mainly enriched in pathways such as “endocytosis”, “plant hormone signal transduction”, “spliceosome”, and “mRNA surveillance pathway” (Figure 6B). 

In the blue, green, and light cyan modules, ten, ten, and six hub genes were identified, respectively (Figure 6C). Notably, isoform_31657 and isoform_174971 in the blue and light cyan modules were recognized as EIL and MYB transcription factors. Pfam domain annotation revealed that the blue module encompassed gene families such as *Ribosomal_S5_C*, *CBFB_NFYA*, *Homeobox_KN*, and *NAP*. For the green module, hub genes belonged to *NYN*, *FH2*, *EF_TS*, and *CBS* gene families. Meanwhile, the light cyan module featured hub genes in the *MYB*, *CBM49*, *RRM_1*, *XS*, and *RRM_1* gene families (Table S3).

### 2.7. Correlation Analysis between SSR Polymorphism and Gene Expression

Analysis of covariance (ANCOVA) analysis revealed that 264 polymorphic genic-SSRs exhibited a significant linear correlation (*p* < 0.05) with the gene expressions of their corresponding transcripts. Among these, the allele length of 161 polymorphic genic-SSRs showed a positive correlation with gene expression levels, while 103 displayed a negative correlation. Moreover, significant quadratic and cubic correlations were identified between SSRs’ length variations and gene expressions in 48 and 34 polymorphic genic-SSRs, respectively (*p* < 0.05) (Figure S3).

**Figure 7:**
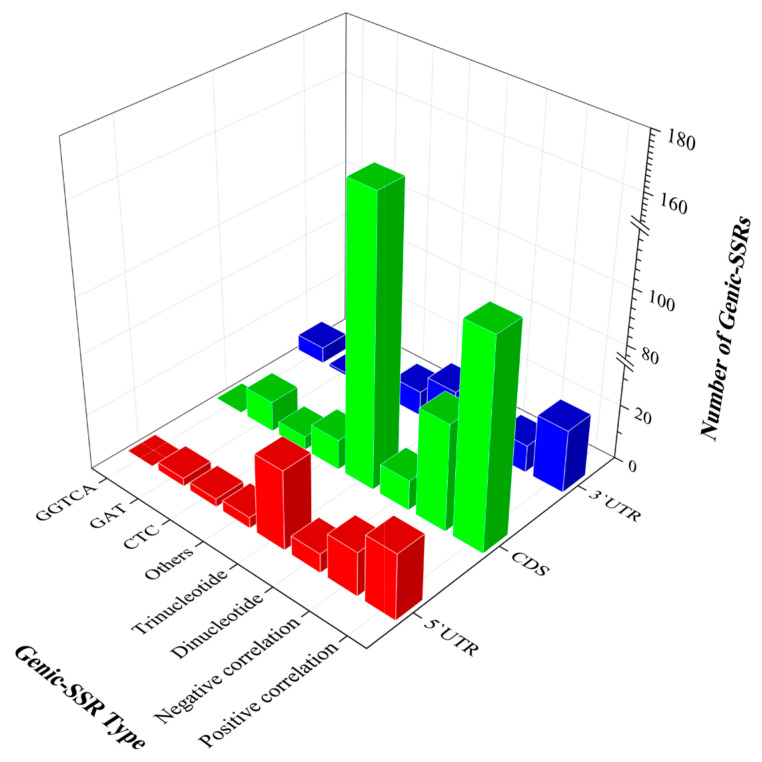
Location of genic-SSR related to gene expression. The *x*-axis represents genic-SSRs’ type, the *y*-axis represents the distribution of the genic-SSRs, the *z*-axis represents the number of genic-SSRs.

Trinucleotides (81.06%) were the most prevalent among these genic-SSRs, primarily distributed in the CDS regions of the SSR-related genes (72.35%). Across 5′UTRs, CDSs, and 3′UTRs, the most abundant motif types were CTC (7.5%), GAT (5.76%), and GGTCA (18.18%), respectively (Figure 7).

**Figure 8:**
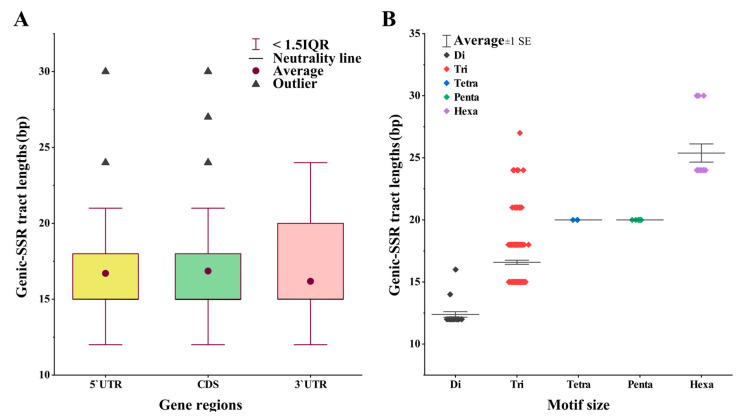
Length variations among genic-SSRs in different gene regions and SSR types. (**A**) Variability in genic-SSR length across gene regions of 5′UTR, CDS, and 3′UTR. (**B**) Variability in genic-SSR length based on microsatellite motif size.

The average length of polymorphic genic-SSRs influencing gene expression was 16.75 bp. There was no significant difference in the mean length of polymorphic genic-SSRs located in the 5′UTRs, CDSs, and 3′UTRs (KW test, *p* = 0.37) (Figure 8A). However, among different nucleotide types, the lengths of the genic-SSRs exhibited a significant difference (KW test, *p* = 0.01) (Figure 8B).

### 2.8. Experimental Validation of SSR Polymorphism and Their Effect on Gene Expression

Out of the 20 selected genic-SSRs, 19 were successfully amplified, and 16 exhibited polymorphic loci (Table S4). The polymorphic nature of these loci was further evaluated through capillary electrophoresis (Figure S4C). The sequencing results (Figure S4B) indicate that the observed polymorphism in the SSRs align with the transcriptome detection results (Figure S4A).

**Figure 9:**
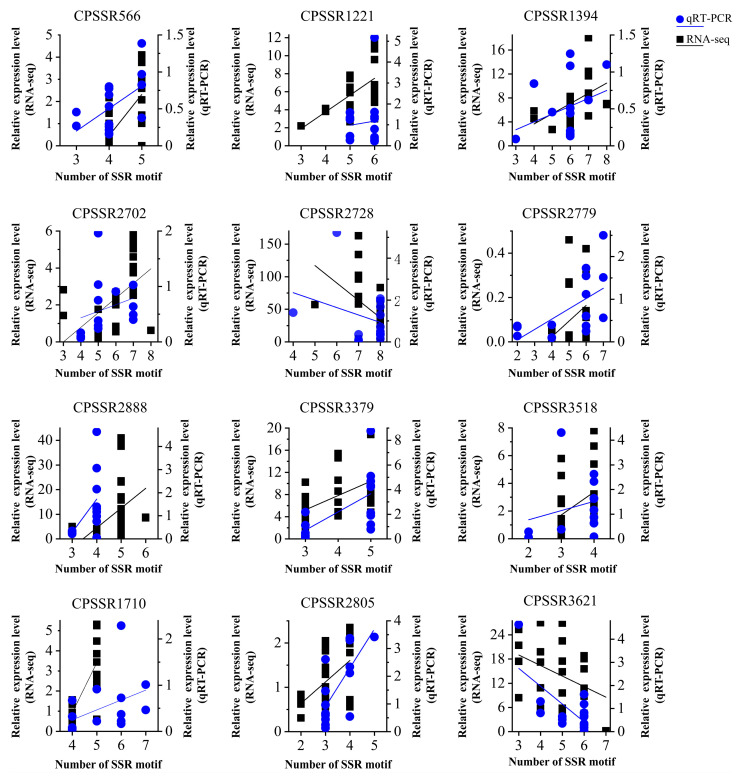
Correlation map of SSR repeat unit number and allele expression. The *x*-axis represents the number of SSRs’ alleles, the *y*-axis represents related gene’s expression.

To validate the linear correlation between SSR allele length and gene expression, sixteen genes containing polymorphic SSRs were selected for qRT-PCR analysis. The results reveal that the expressions of 12 polymorphic SSRs were significantly (*p* < 0.05) correlated with the expressions of their respective genes, and this trend is consistent with the findings from transcriptomic analysis (Figure 9).

## 3. Discussion

### 3.1. Polymorphic Genic-SSRs Provide the Genetic Basis for the Evolution of Ecological Adaptability of Caragana

SSRs are widely distributed repetitive sequences in the genome and are genomic components involved in the formation of biological adaptability. Under selective pressure, the frequency of SSRs in coding regions is lower compared to other genome regions. Studies have shown that SSRs are non-randomly distributed in both gene CDSs and UTRs, potentially impacting gene function and gene products [38,39]. In model plants, such as *Arabidopsis thaliana*, *Oryza sativa*, *Zea mays*, *Glycine max*, *Hordeum vulgare*, and *Triticum aestivum*, trinucleotide repeat SSRs often appear in CDSs [29,40,41], possibly due to the variation in this repeat sequence not causing a significant change in the protein reading frame, resulting in a frame-shift mutation and altering gene expression products to different proteins. In the transcriptome of 12 *Caragana* species, positional analysis of polymorphic genic-SSRs revealed that 72.87% of trinucleotide repeats were located in CDSs, and 31.30% of dinucleotide repeats were distributed in 3′UTRs, aligning with the observations made in other plant transcriptomes [42]. Additionally, genic-SSRs exhibit nucleotide composition bias [38], Similar to *Arabidopsis*, in *Caragana*, AG/CT was the most abundant dinucleotide repeat sequence in gene untranslated regions, predominantly enriched in 5′UTRs, while AT/AT repeats were more prevalent in 3′UTRs; repeat types such as ATC/GAT, AGG/CCT, and AGC/GCT had higher frequencies in CDSs [43].

SSR polymorphism is generally caused by slippage during the replication process [44]. The length variation of SSRs reflects the activity of SSRs in expansion or contraction repetitive sequences. SSR variation in gene sequences increases gene instability, thus providing impetus for gene functional variation [45]. Transcriptomic studies in *Camellia sinensis* have shown that gene expression levels without microsatellite sequences were significantly higher than those containing microsatellite sequences, with genes containing complex microsatellite sequences exhibiting the lowest expression levels, revealing a negative correlation between gene expression and microsatellite length [46]. In salt-stressed *Tilapia*, a negative correlation was observed between microsatellite length and gene expression, whereas the opposite trend was noted under non-stress conditions [12]. In yeast, there was a multiple linear relationship between microsatellite length in promoters and gene expression. Consistent with existing reports [10,47], the present study identified 346 polymorphic genic-SSRs that significantly affect the expression of related genes. In both gene coding and untranslated regions, the variations of 48 and 32 genic-SSRs in *Caragana* showed quadratic or cubic correlations with the expression of related genes (*p* < 0.05). This suggests that the low-level expression of these genes in specific species may relax selective pressure, retaining these genes in the genome.

The position of polymorphic SSRs in genes may also affect the expression of the gene. Numerous lines of evidence have shown that 5′UTRs-SSRs have a significant impact on gene expression [12,48,49]. They achieve this by altering cis-regulatory elements, causing changes in transcription factor binding sites, thereby regulating gene expression [48]. For example, variations in the (CT)_n_ repeat sequence in the 5′UTR of *Catharanthus roseus* can regulate the promoter activity of the *tryptophan decarboxylase* (*TDC*) gene [48]. In the promoter region of the heat shock protein gene *HSP26* in *Drosophila melanogaster*, *Aspergillus*, and *Phytophthora infestans*, the (TC)_n_ repeat plays a role in transcription factor function [50,51,52]. Variation in coding-region polymorphic SSRs may be due to changes in protein structure, including transcription factors [53,54,55]. The instability of microsatellites in the coding region of senescent *A. thaliana* is due to the frequent involvement of the nonhomologous end-joining repair pathway (MHEJ) in DNA repair, affecting DNA polymerase activity [56]. In this study, 434, 2355, and 499 genes contained 440, 2409, and 508 SSRs in 5′UTRs, CDSs, and 3′UTRs, respectively. Functional annotation indicated that these genic-SSR-related genes were mainly enriched in GO terms such as “binding”, “cellular processes”, and “intracellular”, as well as in KEGG pathways like “secondary metabolite synthesis”, “spliceosome”, and “plant hormone signaling”. The results suggest that the variations of these SSRs may regulate such cellular processes and metabolic pathways, acting as genomic hotspots in the formation of ecological adaptability in *Caragana* plants. Among the polymorphic genic-SSRs linearly correlated with gene expression, 15.15% were located in the 5′UTR, and 72.35% were in the CDS. This suggests a regulatory role similar to that shown previous studies. Additionally, in *Caragana*, there was no significant difference in the average length of SSRs linearly correlated with gene expression in 5′UTR, CDS, and 3′UTR. This indicates that the expansion or contraction of the SSRs might be a random process, independent of the gene region they were located [10].

### 3.2. Potential Roles of Polymorphic Genic-SSRs in the Formation of Ecological Adaptations in Caragana

Phenotypic variation is the direct evidence of plant response to climate change and is an intuitive manifestation of genotype interacting with environmental changes, providing insights into the direct effects of environmental pressures on plants. Heterogeneous environments are a major factor promoting phenotypic evolution [57,58], closely related to the formation of plant phenotypic diversity [59,60,61,62]. The correlation analysis between climatic factors in different habitats and WGCNA gene modules revealed that genes in *Caragana*-specific modules may play a crucial role in responding to different hydrothermal conditions in diverse habitats. Numerous studies have indicated that seasonal and interannual climate fluctuations are dominant factors driving plant phenotypic variation [58,63], especially variations driven by temperature and precipitation changes [58]. The 12 *Caragana* species investigated in this study are widely distributed across Inner Mongolia, Tibet, and Xinjiang, with significant differences in elevation and hydrothermal conditions among the sampling sites, contributing to the diversity of functional traits in this plant genus [18].

Plant functional traits are adaptive characteristics developed through long-term evolution in response to environmental interactions. The variation in SSRs within the transcriptome can influence gene function, subsequently leading to changes in corresponding traits [64,65]. Gene co-expression network analysis revealed significant correlations between *Caragana* functional traits and polymorphic genic-SSR-associated gene modules. Genes in these modules were enriched in GO terms related to “biological regulation”, “binding”, “cellular components”, and pathways such as “plant hormone signaling”, “ribonucleic acid surveillance”, and “spliceosome”. These results indicate their role as pioneer genes in *Caragana*’s response to complex climate factors. Therefore, SSR variation plays a role in facilitating changes in different *Caragana* functional traits, contributing to diverse ecological adaptations of the genus. Particularly, 26 core genes in these modules, with regulatory roles and significant correlations with climate factors, have been demonstrated to play crucial roles in regulating plant responses to environmental stress, including transcription factor-encoding genes such as *MYB*, *TCP*, *HSP90*, and *ARF*, as well as genes from the *HECT* and *CBS* families.

Lignin, as a crucial component of the secondary cell wall in vascular plants, enhances plant resistance to environmental changes and mechanical damage by increasing cell wall mechanical strength and stem hardness. MYB transcription factors participate in the regulation of lignin secondary metabolism. In *A. thaliana*, MYB20, MYB42, and MYB43 activate the expression of genes related to lignin synthesis, mediating secondary wall formation [66]. In *Z. mays*, the *MYB167* gene [67] and in *Populus tomentosa*, the *MYB216* gene [68] are involved in lignin biosynthesis. In this study, the *MYB* gene (isoform_174971) in the light cyan module positively regulated *Caragana* stem tissue density (STD), suggesting its involvement in the biosynthesis of lignin in *Caragana* plants. TCP transcription factors participate in plant stem elongation. In *A. thaliana* [69], Class I TCP transcription factors directly bind to cell cycle-related genes. DELLA proteins, as negative regulators in the gibberellin (GA) signaling pathway, can inhibit TCP activity, and mutants with *tcp8-tcp14-tcp15-tcp22* exhibit extreme dwarfism and are not responsive to GA regulation. These results indicate that Class I TCPs can regulate *Arabidopsis* plant height by mediating the GA signal. The *Arabidopsis* AtTCP4 transcription factor regulates the *YUCCA5* gene, a key enzyme in auxin synthesis, and plays a crucial role in the elongation of the embryonic axis under light and auxin regulation [70]. The *T. aestivum HECT* gene shows high expression levels in the stem during different developmental stages and the 1cm spike stage, suggesting its role in regulating wheat stem elongation [71]. In this study, *TCP* transcription factor genes and *HECT* genes were core genes in the purple module, potentially playing a crucial role in regulating *Caragana* plant height traits. The *O. sativa ARF1* gene plays a significant role in regulating plant growth, leaf size, and plant height [72]. *OsARF1* interacts with *OsIAA1*, affecting plant endogenous auxin levels, thereby regulating rice leaf angle [73]. *OsARF19* is involved in regulating leaf angle size, and when overexpressed, the leaf lobes near the axis cells overextend, leading to an increase in the leaf angle [74]. In this study, *ARF* transcription factor genes in the turquoise module collectively regulate *Caragana* crown coverage (CC) traits along with other core genes. *SLAC1*, encoding an anion channel, is expressed in guard cells [75,76], mediating Cl^-^ and NO_3_^-^ efflux, causing guard cell dehydration and stomatal closure [77]. Under drought conditions, plants often close some stomata to reduce internal water loss. Stomatal closure can lead to a decrease in intercellular CO_2_ concentration, weakening plant photosynthesis, and an increase in respiratory consumption, resulting in a decrease in plant carbon biomass (Cmass). The number and size of cells can affect the size and characteristics of plant tissue organs. The F-box domain-containing gene *AtFBX92* in *A. thaliana* inhibits leaf growth [78]. Trait correlation analysis in soybean (*Glycine max*) reveals a significant positive association between leaf width and CC [79]. This aligns with our study’s observations, wherein *F-box* genes in the turquoise module exert inhibitory effects on *Caragana* CC traits.

The CBS domain in plants plays a role in defending against low-temperature stress, as seen in genes like *OsCBSX2*, *OsCBSX9*, *OsCBSCLC4*, *OsCBSCLC5*, and *OsCBSDUF1* in rice, which exhibit high expression levels under cold conditions [80]. In wheat, the *TaCBS-10s* gene has the highest expression level at −10 °C, slightly decreasing at −25 °C, suggesting that its cold resistance function is relatively weak [81]. In this study, the isoform_272480 gene with a CBS domain in the green module was significantly negatively correlated with the BIO2. This indicates that it was influenced by BIO2 and plays a role as a core gene in the green module, regulating *Caragana*’s adaptation to temperature differences in different habitats. The Fasciclin domain can affect plant cell wall formation by regulating the distribution and deposition of cellulose in the cell wall [82,83]. The overexpression of the rice *rFCA* gene, containing RRM_1 and RRM_2 domains, participates in the regulation of transgenic rice plant cell size [84]. In this study, genes in the salmon module, including *Fasciclin* and *RRM_1*, show significant correlations with *Caragana* traits, such as plant height (H), crown coverage (CC), leaf dry mass (LDM), leaf wet mass (LWM), stem diameter (SD), total leaf area (TLA), and leaflet area (LA).

In conclusion, combining the identified impact of genic-SSRs on gene expression and their roles in *Caragana* phenotypic variation and response to environmental changes, it can be inferred that unstable SSRs in *Caragana* genes are essential driving factors for their ecological adaptation diversity in variable environments.

## 4. Materials and Methods

### 4.1. Plant Materials

**Figure 10:**
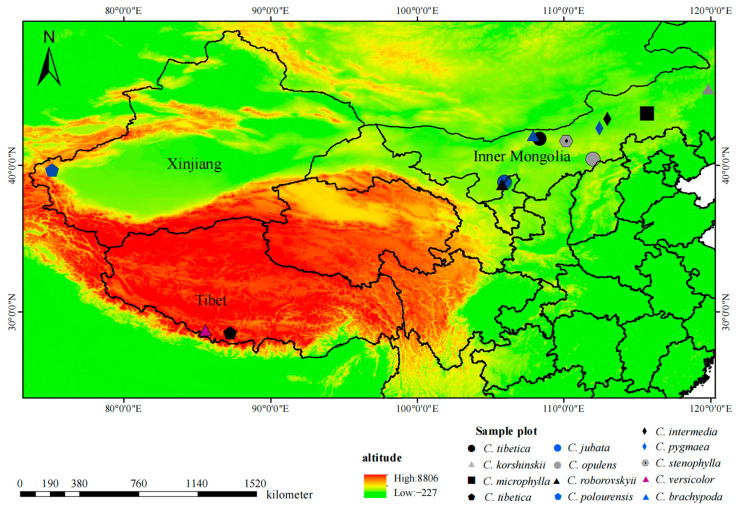
The geographical locations of the 12 *Caragana* species investigated in this study.

Fresh leaves and seeds were collected from 12 *Caragana* species across diverse regions, including Inner Mongolia, Tibet, and Xinjiang, China. Specifically, *C. polourensis* samples were obtained from the mountainous regions of Xinjiang, while *C. versicolor* and *C. tibetica* (Tibet) were sourced from the Tibetan plateau areas. The remaining species were collected in Inner Mongolia, encompassing various habitat types. Notably, the sampling locations in Inner Mongolia exhibited a distinct geographical substitution distribution along the environmental gradient, extending from the low mountains and hills in the east, central grassland area westward to the desert region. This coverage spanned a geographical range of 38.67–45.24° N, 105.80–119.84° E, with elevations ranging from 1058 to 3472 m. Of the samples, *C. korshinskii* was collected from the southern mountains of Daxing’an Mountains in the East, *C. pygmaea*, *C. intermediate*, *C. stenophylla* and *C. brachypoda* were collected from the vast the Inner Mongolian Plateau, *C. microphylla* was collected near the Hunshandak Sandy Land, while *C. opulens* were collected in the northern mountains of Lvliang Mountain. Additionally, *C. jubata* and *C. roborovskyii* were sampled from Helan Mountain (Figure 10). In each sample plot, 3–5 individuals with robust growth were randomly selected at 30 m intervals for the sampling process. Subsequent to collection, the leaf samples were rapidly frozen in liquid nitrogen and stored in a −80 °C refrigerator until transportation to the laboratory. Detailed geographical details of the sampling sites are provided in Table 1. 

### 4.2. Full-Length Transcriptome Sequencing and Bioinformatics Analysis

Fresh leaves of *C. korshinskii* were used for RNA extraction, employing TRIzol reagent (Invitrogen, Carlsbad, CA, USA) following the provided instructions. Subsequently, the RNA sample was treated with deoxyribonuclease I (TaKaRa Bio Inc., Otsu, Shiga, Japan) for 30 min at 37 °C to remove residual DNA. Quantification and quality assessment of the total RNA were performed using the Agilent 2100 Bioanalyzer (Agilent Technologies, Palo Alto, CA, USA), ensuring a minimum RNA integrity number of 6.5. The RNA sample was then reverse-transcribed using the UMI base PCR cDNA Synthesis Kit to generate high-quality first-strand FL cDNA. First-strand FL cDNAs exceeding 4 kb were chosen for the synthesis of the second-strand cDNA. PCR products underwent purification by AMPure PB Bead, and the SMRTbe II library was constructed. The PacBio Sequel™ sequencing platform was employed to sequence the full-length transcriptome. SMRT Link (v5.0.1) was utilized for analyzing raw sequencing data, clustering transcripts, and extracting circular consensus sequence (CCS) from subreads.

The CCSs were categorized into short sequences, chimeras, non-full-length reads (nFL reads), and full-length non-chimeric reads (FLNC reads) based on the presence of primer sequences and poly (A) tail in the CCSs. After clustering, correcting, and filtering the FLNC reads using the Arrow algorithm and ICE algorithm, high-quality isoforms were obtained. Subsequently, de-redundancy was performed using cd-hit (v4.6.8) to derive unique isoform sequences (UniqIsoforms). 

Functional annotation involved aligning the UniqIsoforms with public databases, including the non-redundant (NR) protein database, the Swiss-Prot protein database, the Kyoto Encyclopedia of Genes and Genomes (KEGG) pathway database, and the euKaryotic Orthologous Groups (KOG) database. Gene Ontology (GO) functional categories were identified using Blast2GO software (v2.5.0), and the Nucleotide (NT) sequence database was screened by Blastn.

TransDecoder (v3.0.1) was employed to search for the open reading frame (ORF) of the full-length isoforms and extract long reading frame sequences. Based on sequence similarity, the isoforms were mapped to the Swissprot database, and the blast results were screened in the Pfam database by Hmmscan. Finally, TransDecoder was used to predict the CDS and UTR of the isoforms.

### 4.3. Short-Reads Transcriptome Sequencing and Bioinformatics Analysis

RNA was extracted from the fresh leaves of three individuals from each of the 12 *Caragana* species using the aforementioned RNA isolation and purification protocol. Subsequently, poly(A) mRNA was isolated with Oligo(dT) beads and used for constructing cDNA libraries. In summary, purified mRNA underwent fragmentation, followed by the generation of first-strand cDNA using random hexamer-primed reverse transcription and second-strand cDNA synthesis. A-Tailing Mix and RNA Index Adapters were added for end repair. The resulting cDNA fragments were then amplified by PCR and purified using Ampure XP Beads. Subsequently, the double-stranded PCR products were denatured and circularized with a splint oligo sequence to obtain single-strand circular DNA (ssCir DNA). The ssCir DNAs were amplified with phi29 polymerase to form DNA nanoballs (DNBs) and generate the library. Finally, the cDNA library for each sample underwent paired-end sequencing using the BGISEQ platform (BGI Tech Solution Co., Ltd., Wuhan, China).

After sequencing, adaptor reads, low-quality reads, and reads in which the proportion of “N” was greater than 5% were filtered to obtain clean reads. The clean reads were assembled using Trinity (v2.0.6). The assembled transcripts were then clustered using Tgicl (v2.1) for redundancy to obtain unigenes for each sample. To assess the integrity of the assembled transcriptome, the assembled transcripts were evaluated for quality using conserved sequences in the Single-Copy Orthologs database Benchmarking Universal Single-Copy Orthologs (BUSCO).

The clean reads of each sample were mapped to the full-length *C. korshinskii* transcriptome using Bowtie2 (v2.2.5) [85], and the mapped reads of each sample were quantified using RSEM (v1.2.8) [86]. The expression levels of the genes were normalized using fragments per kilobase of exon model per million mapped fragments (FPKM).

### 4.4. Identification and Characteristic Analysis of Polymorphic Genic-SSRs

Using the full-length transcriptome as a reference, we utilized CandiSSR [87] to identify potential polymorphic genic-SSRs within the assembled unigene datasets of the 12 *Caragana* species. The minimum number of SSR repeats for di-, tri-, tetra-, penta-, and hexa-nucleotide repeats was set at six, five, five, four, and four, respectively. The length of sequences on both sides of the SSR loci was required to be more than 100 bp. The BLAST E-value was set at 1.0 × 10^−10^, and the similarity and coverage were set at 95%.

The location of genic-SSRs within the isoform was determined based on the relative positions of the SSRs concerning the start (ATG) and stop (TAA, TAG, and TGA) codons of the transcript. To gain insights into the functional information of genic-SSR-containing isoforms, GO functional categories were identified using Blast2GO software (v2.5.0). Additionally, pathways were assigned through sequence searches against the KEGG database using the BLASTX algorithm, with an e-value threshold set at 1.0 × 10^−5^.

### 4.5. Weighted Gene Co-Expression Network Analysis and Hub Gene Identifying

Based on quantitative gene expression results, DEGs were identified using DESEQ2 with a threshold of |log_2_foldchange| ≥ 1 and a *q*-value ≤ 0.05. Subsequently, DEGs were filtered based on a mean FPKM in all samples greater than 1. Genic-SSR-containing DEGs were then isolated. Utilizing these selected genes as input, the WGCNA package was applied to determine the soft threshold and estimate the optimal weight value. Initially, β values ranging from 1 to 30 were tested to calculate the correlation coefficient and mean gene connectivity. The selection criterion for β aimed to achieve a correlation coefficient squared close to 0.88 while maintaining a reasonable level of gene connectivity. Following this, genes were categorized according to their expression patterns, and the dynamic shearing algorithm was used to group genes with similar expression patterns into distinct modules.

The modules were subsequently correlated with functional traits and climate factors of the sampling locations for the studied *Caragana* species, identifying modules with high correlation (|r| ≥ 0.65). Functional traits encompassed various parameters, including height (H), canopy coverage (CC), leaf thickness (LT), leaf dry mass (LDM), leaf wet mass (LWM), leaf dry mass content (LDMC), specific leaf area (SLA), stem volume (SV), stem diameter (SD), stem dry weight (SDW), stem tissue density (STD), total leaf area (TLA), leaf area (LA), stable carbon isotope 13C (δ13C), carbon biomass (Cmass), and nitrogen biomass (Nmass). These functional trait data were sourced from measurements conducted by Luo et al. [18]. Climate factor data, such as longitude, attitude, altitude, annual mean temperature (BIO1), mean diurnal range (BIO2), isothermality (BIO3), temperature seasonality (BIO4), max temperature of the warmest month (BIO5), min temperature of the coldest month (BIO6), temperature annual range (BIO7), mean temperature of the wettest quarter (BIO8), mean temperature of the driest quarter (BIO9), mean temperature of the warmest quarter (BIO10), mean temperature of the coldest quarter (BIO11), annual precipitation (BIO12), precipitation of the wettest month (BIO13), precipitation of the driest month (BIO14), precipitation seasonality (BIO15), precipitation of the wettest quarter (BIO16), precipitation of the driest quarter (BIO17), precipitation of the warmest quarter (BIO18), and precipitation of the coldest quarter (BIO19), were obtained from the WorldClim database (https://www.worldclim.org/, accessed on 27 October 2022).

To identify the functional information of DEGs in specific modules, genes within these modules were categorized using the GO and KEGG databases for functional classification. Enrichment analysis was conducted using the phyper function in R (v4.2.1).

Hubgenes within specific modules were identified by visualizing the interaction network among genes using Cytoscape (v3.9.1) software, focusing on high weight node relationships within the particular module. Hubgenes within the module were selected based on their high degree in the network. Transcription factor information for the predicted hubgenes was obtained by cross-referencing the PlantTFDB database with the module’s hubgenes. Furthermore, Hmmer was employed to annotate the protein domain of hubgenes in the Pfam database, providing insights into the functions of these hubgenes.

### 4.6. Effect of Genic-SSRs’ Allele Length on Gene Expression

The effect of the length of polymorphic genic-SSRs alleles on gene expression was examined using ANCOVA. A *p*-value of 0.05 was employed to identify genic-SSRs where the allele length significantly correlates with gene expression. The *p*-values obtained from the linear model were adjusted for multiple comparisons, with a *q*-value of 0.05 serving as the significance threshold [88]. Linear, quadratic, and cubic regression models were all attempted, as previous in vivo experiments have identified nonlinear relationships between microsatellite allele length and gene expression [89]. Additionally, we conducted a Kruskal–Wallis test to analyze the length, type frequency, and location of polymorphic genic-SSRs associated with gene expression.

### 4.7. Validation of SSR Polymorphism and Their Effect on Gene Expression

To validate genic-SSR polymorphism and its impact on the expression of associated genes, twenty SSR loci were selected for experimentation. Primers used to amplify the SSR loci were designed using the NCBI Primer-Blast program. Genomic DNA was extracted from 40 individuals of 5 *Caragana* species (*C. korshinskii*, *C. microphylla*, *C. intermedia*, *C. versicolor*, *C. brachypoda*) grown indoors, utilizing CTAB. The DNA quality was assessed on 1% TAE agarose gels and NanoDrop 2000c (Gene Company Limited, Beijing, China). PCR amplifications were carried out on the ABI2720 Thermal cycler in 25 µL reaction mixtures containing 1 µL of template DNA (50 ng/µL), 12.5 µL of Premix Taq (TaKaRa Biotechnology Co., Dalian, China), 0.5 µL (10 pM) of forward primer, 0.5 µL (10 pM) of reverse primer, and ddH_2_O. PCR amplifications proceeded as follows: 5 min at 9 °C, followed by 30 cycles of 30 s at 94 °C, 30 s at the primer-specific annealing temperature, 30 s at 72 °C, and a final extension step at 72 °C for 10 min. For successfully amplified primer pairs, the 5′ end of each forward primer was tagged with one of three fluorescent dyes (6-carboxy-fluorescine, hexachloro-6-carboxy-fluorescine, and 6-carboxy-X-rhodamine) and used for amplifications with the same protocol. The labeled PCR products were analyzed on an ABI 3730xl DNA analyzer with a GeneScan^TM^ 500 LIZ^TM^ Size Standard (Applied Biosystems, Beijing, China). Additionally, PCR products were sequenced to identify the repeat number of a genic-SSR.

For gene expression analysis, the total RNAs were isolated from the above samples using the Eastep^®^ Super Total RNA Extraction Kit (Promega, Shanghai, China). Then, 1 μg total RNA was reverse-transcribed into cDNA using a PrimeScript RT Reagent Kit with genomic DNA (gDNA) Eraser (TaKaRa, Dalian, China). Fourteen genic-SSRs that exhibited a linear correlation with the gene expression of relevant genes were selected for qRT-PCR validation, which was performed on a real-time PCR platform (BioRad, Hercules, CA, USA). Reactions were carried out in a 20 μL final volume, containing 100 ng of cDNA template, 0.8 μL (0.4 um) each of forward and reverse primers, 10 μL of SYBR Premix Ex Taq II (TaKaRa, Dalian, China), 0.4 μL of ROX Reference Dye or Dye II, and 6 μL of sterile distilled water. The thermal cycling programs were as follows: initial denaturation at 95 °C for 30 s, followed by 40 cycles of denaturation at 95 °C for 5 s, and annealing and extension at 60 °C for 34 s. All reactions were performed with three technical replicates across the nine unigenes. The relative expression levels of the investigated genes were normalized to *actin-7* (isoform_222161) and calculated using the 2^−ΔΔ^Ct method.

Using a *p*-value of 0.05 as the significance threshold, the correlation between the number of polymorphic SSR loci and the relative expression of alleles was analyzed using Origin 2021 Software to verify whether there was a linear correlation between SSR variation and gene expression.

## 5. Conclusions

Genic-SSRs are crucial molecular regulatory elements contributing to the diversity of gene functions within plant genomes. In the present study, we identified genic-SSRs in the transcriptomes of 12 *Caragana* species in China and analyzed their types, frequencies, and locations. We observed that a subset of genic-SSR-associated genes was significantly linked to the functional traits and climate factors of the studied *Caragana* plants. Subsequently, we discussed the potential roles of these genes in shaping the evolutionary mechanisms of ecological adaptation within the genus. Our findings not only provide novel candidates for further studying *Caragana*’s adaptation mechanisms based on gene expression and genetic variation but also contribute to a broader understanding of genic-SSRs in plants. In subsequent studies, we will explore more genic-SSR polymorphisms at both individual and species levels in *Caragana*, conduct experiments to explore the functions of specific polymorphic genic-SSRs, and identify how genic-SSRs drive environmental adaptation in the genus.

## Figures and Tables

**Table 1:** Habitat information of 12 species of *Caragana*.

Species	Longitude (E)	Latitude (N)	Altitude (m)
*C. tibetica* (Inner Mongolia)	108°19′09.63″	41°48′00.61″	1411
*C. korshinskii*	119°50′38.87″	45°14′05.92″	1149
*C. microphylla*	115°42′07.48″	43°31′48.47″	1191
*C. tibetica* (Tibet)	87°14′02.1″	28°36′15.9″	4231
*C. jubata*	105°56′53.12″	38°50′09.92″	3472
*C. opulens*	112°00′35.78″	40°25′27.07″	1906
*C. roborovskyii*	105°48′00.58″	38°40′16.90″	2004
*C. polourensis*	75°04′05.9″	39°42′22.1″	2130
*C. intermedia*	112°58′10.87″	43°11′22.24″	1058
*C. pygmaea*	112°25′54.79″	42°34′1.61″	1213
*C. stenophylla*	110°10′16.62″	41°40′41.93″	1573
*C. versicolor*	85°32′48.6″	28°45′35.1″	4625
*C. brachypoda*	107°54′59.61″	42°02′46.33″	1310

## Data Availability

The data presented in this study are still undergoing further analysis, it is available upon request from the corresponding author.

## Supplementary Materials

The following supporting information can be downloaded at: https://www.mdpi.com/article/10.3390/ijms25042084/s1.
