# Supplementary material for: The Potential Role of Genic-SSRs in Driving Ecological Adaptation Diversity in Caragana Plants
Source: Int J Mol Sci. 2024 Feb 8;25(4):2084. doi: 10.3390/ijms25042084 (PMC10888960; doi:10.3390/ijms25042084)
Supplement: Supplementary file 1 [file ijms-25-02084-s001.zip › Figure S1.pdf]

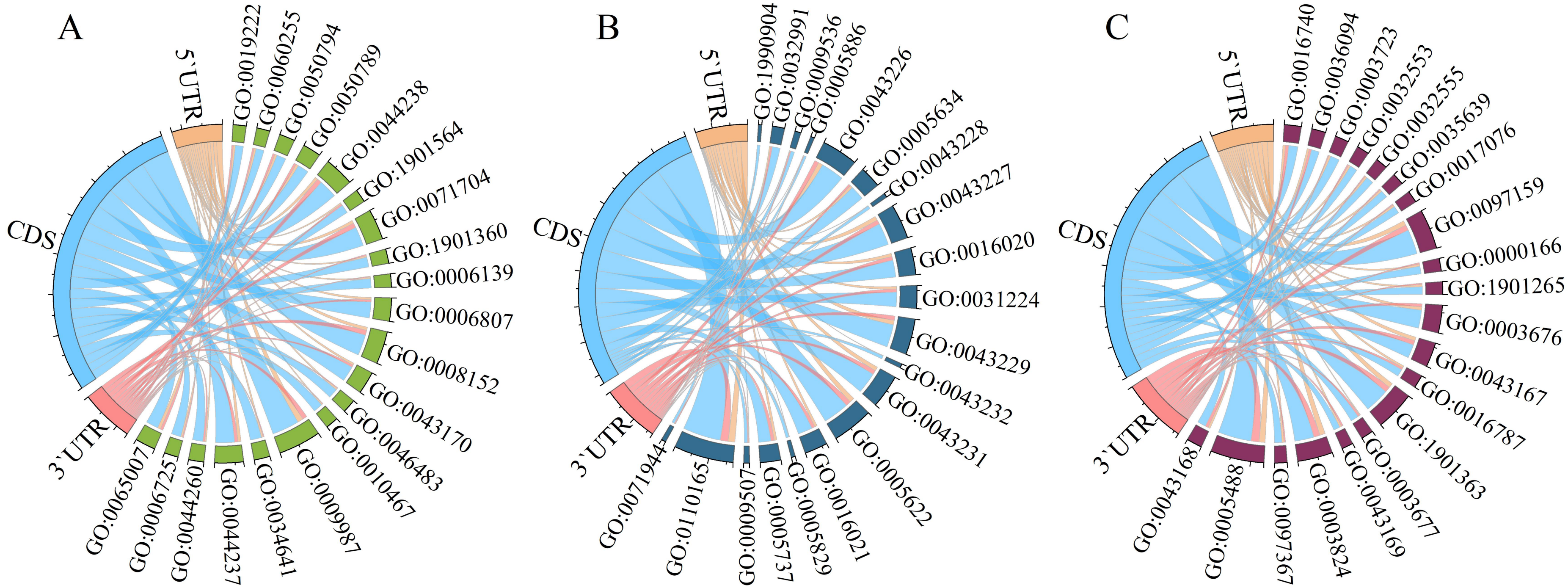

Figure S1. Go functional annotation of genes containing polymorphic Genic-SSRs. (**A**–**C**) represent the annotation results in the three main GO categories, i.e., biological process, cellular component, and molecular function.
