# Supplementary material for: The Potential Role of Genic-SSRs in Driving Ecological Adaptation Diversity in Caragana Plants
Source: Int J Mol Sci. 2024 Feb 8;25(4):2084. doi: 10.3390/ijms25042084 (PMC10888960; doi:10.3390/ijms25042084)
Supplement: Supplementary file 1 [file ijms-25-02084-s001.zip › Figure S2.pdf]

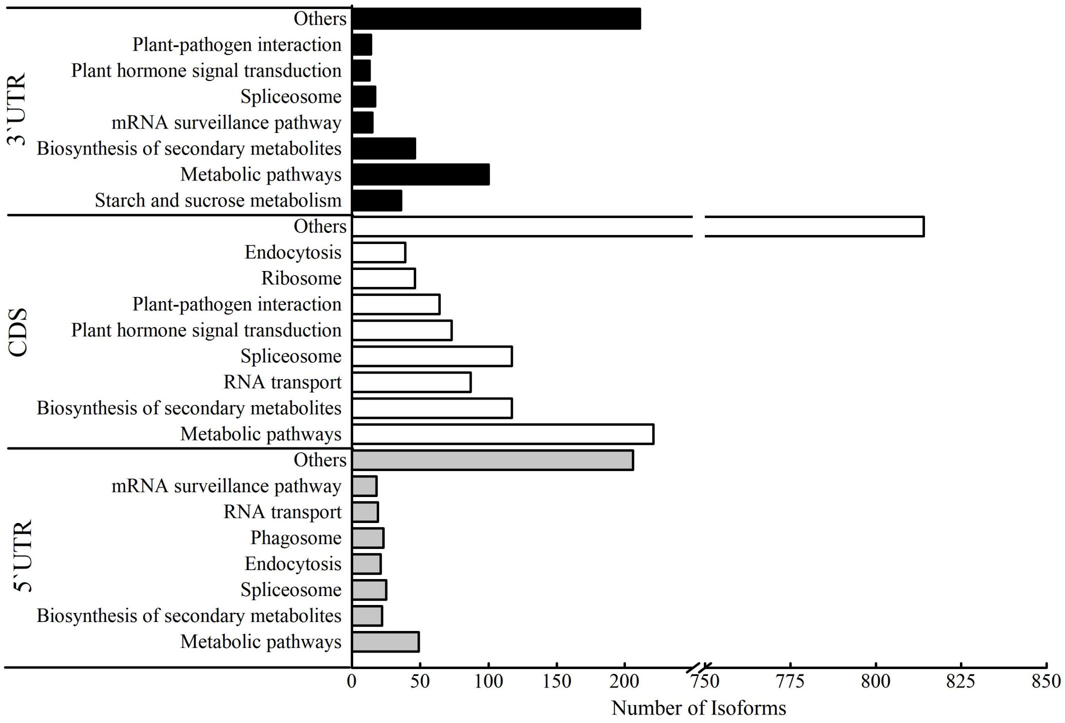

Figure S2. KEGG functional annotation results of gene sequences containing polymorphic Genic-SSRs loci. The x-axis represent the numbers of SSR-containing sequences enriched in KEGG pathways, the y-axis represent the enriched pathways assigned to the 5′UTR, CDSs, and 3′UTRs SSR-containing sequences.
