# Supplementary material for: The Potential Role of Genic-SSRs in Driving Ecological Adaptation Diversity in Caragana Plants
Source: Int J Mol Sci. 2024 Feb 8;25(4):2084. doi: 10.3390/ijms25042084 (PMC10888960; doi:10.3390/ijms25042084)
Supplement: Supplementary file 1 [file ijms-25-02084-s001.zip › Figure S3.pdf]

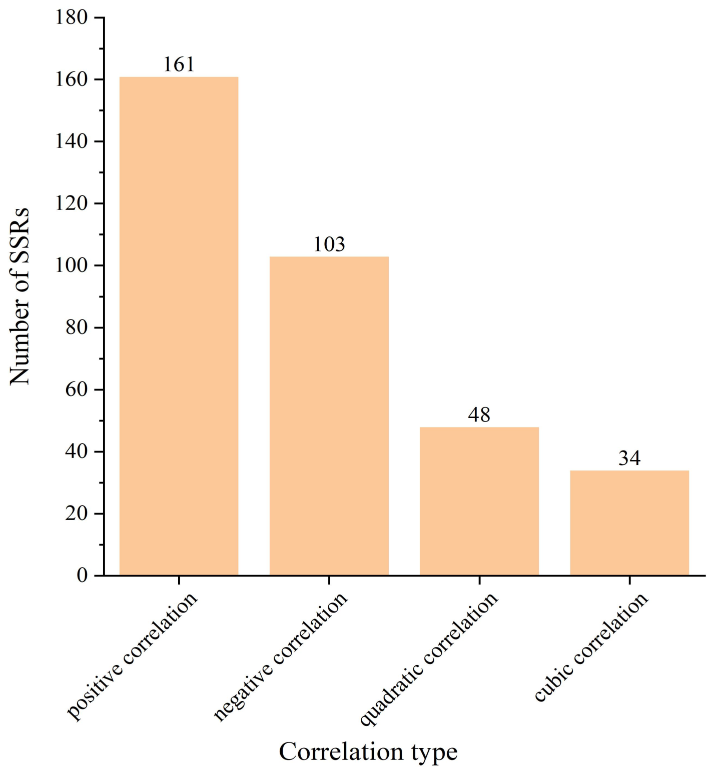

Figure S3. Correlation type quantity chart. The x-axis represents positive correlation, negative correlation, quadratic correlation, cubic correlation, the y-axis represents the number of SSRs loci.
