# Supplementary material for: The Potential Role of Genic-SSRs in Driving Ecological Adaptation Diversity in Caragana Plants
Source: Int J Mol Sci. 2024 Feb 8;25(4):2084. doi: 10.3390/ijms25042084 (PMC10888960; doi:10.3390/ijms25042084)
Supplement: Supplementary file 1 [file ijms-25-02084-s001.zip › Figure S4.pdf]

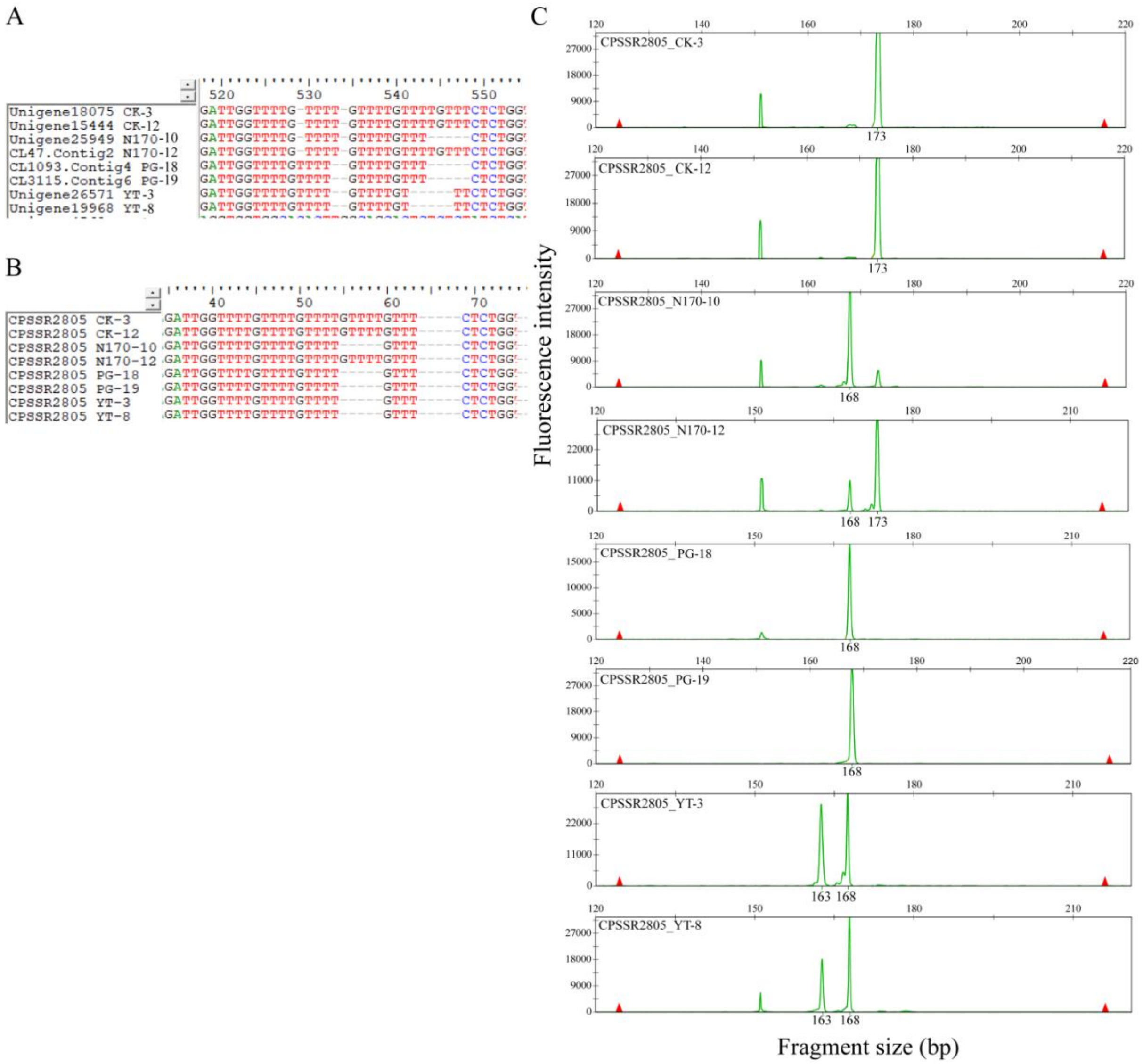

Figure S4. Verification results for CPSSR_2805 (GTTTT). (**A**) multiple sequence alignment of the assembled sequences containing CPSSR_280 5 in the eight *Caragana* transcriptome datasets; (**B**) sequencing results of the PCR products in (**C**); (**C**) the capillary electrophoresis results of CPSSR_2805 in eight *Caragana* individuals.
