# Supplementary material for: The Potential Role of Genic-SSRs in Driving Ecological Adaptation Diversity in Caragana Plants
Source: Int J Mol Sci. 2024 Feb 8;25(4):2084. doi: 10.3390/ijms25042084 (PMC10888960; doi:10.3390/ijms25042084)
Supplement: Supplementary file 1 [file ijms-25-02084-s001.zip › Supplementary Tables.pdf]

Table S1. Sequencing output of thirteen *Caragana* species.

Samples	Raw Reads(M)	Clean Reads(M)	Clean Bases(Gb)	Q20(%)	Q30(%)	Number of Unigene
C. tibetica(IM)_1	42.33	42.33	4.23	98.08	90.97	56,105
C. tibetica(IM)_2	42.33	42.33	4.23	97.84	90.33	63,832
C. tibetica(IM)_3	42.33	42.33	4.23	97.81	90.3	69,322
*C. korshinskii*_1	43.82	42.11	6.32	97.59	93.53	64,058
*C. korshinskii*_2	43.82	42.12	6.32	97.7	93.85	66,741
*C. korshinskii*_3	45.57	42.77	6.41	97.62	93.63	67,649
*C. microphylla*_1	43.82	42.26	6.34	97.55	93.44	69,203
*C. microphylla*_2	45.57	43.6	6.54	97.53	93.42	66,787
*C. microphylla*_3	45.57	42.88	6.43	97.61	93.6	61,667
C. tibetica(TB)_1	42.34	42.34	4.23	97.5	88.97	60,421
C. tibetica(TB)_2	42.34	42.34	4.23	97.38	88.7	63,945
C. tibetica(TB)_3	42.34	42.34	4.23	97.53	88.96	63,882
*C. jubata*_1	43.82	42.09	6.31	97.16	92.72	66,130
*C. jubata*_2	39.49	37.55	5.63	97.12	92.73	59,497
*C. opulens*_1	42.33	42.33	4.23	98.41	91.84	47,372
*C. opulens*_2	42.33	42.33	4.23	98.41	91.84	51,818
*C. opulens*_3	42.33	42.33	4.23	98.05	90.81	66,130
*C. roborovskyii*_1	42.33	42.33	4.23	97.78	90.35	89,740
*C. roborovskyii*_2	42.33	42.33	4.23	97.73	89.95	86,689
*C. roborovskyii*_3	42.33	42.33	4.23	97.99	90.92	87,483
*C. polourensis*_1	42.61	42.61	4.26	98	91.03	53,124
*C. polourensis*_2	42.61	42.61	4.26	97.92	90.68	60,410
*C. polourensis*_3	42.61	42.61	4.26	97.89	90.63	58,969
*C. intermedia*_1	42.33	42.33	4.23	97.87	90.57	76,508
*C. intermedia*_2	42.33	42.33	4.23	97.78	90.23	112,247
*C. intermedia*_3	42.33	42.33	4.23	97.83	90.13	85,253
*C. pygmaea*_1	42.33	42.33	4.23	97.85	90.31	66,621
*C. pygmaea*_2	42.33	42.33	4.23	97.96	90.55	71,544
*C. pygmaea*_3	42.33	42.33	4.23	98.37	91.61	65,407
*C. stenophylla*_1	42.33	42.33	4.23	97.79	89.89	52,692
*C. stenophylla*_2	42.33	42.33	4.23	97.36	88.6	46,457
*C. stenophylla*_3	42.34	42.34	4.23	98.33	91.42	59,649
*C. stenophylla*_1	42.34	42.34	4.23	97.67	89.62	47,146
*C. stenophylla*_2	42.34	42.34	4.23	97.41	89.83	52,942
*C. stenophylla*_3	42.34	42.34	4.23	97.54	89.97	52,508
*C. brachypoda*_1	42.33	42.33	4.23	97.72	89.97	87,097
*C. brachypoda*_2	42.33	42.33	4.23	97.25	89.28	57,667
*C. brachypoda*_3	42.33	42.33	4.23	97.84	90.06	56,365

Table S2. Functional annotation of hubgenes related to functional traits.

Hubgene	SSR Type	Module	Pfam Domain	Functional Annotation
isoform_162345	CCT	green	UPF0016	Function unknown
isoform_219376	TGA	green	PTPlike_phytase	Posttranslational modification, protein turnover, chaperones
isoform_7662	TTAATA	green	Pkinase	Signal transduction mechanisms
isoform_965	TCA	green	NYN	Function unknown
isoform_122092	CTC	green	FH2	Signal transduction mechanisms, Cytoskeleton
isoform_102491	GGT	green	DEAD	RNA processing and modification
isoform_103046	TTC	green	EF_TS	Translation, ribosomal structure and biogenesis
isoform_272480	AGC	green	CBS	Energy production and conversion
isoform_32586	CTA	green	DUF3082	Function unknown
isoform_195328	CAG	green	HhH-GPD	Replication, recombination and repair
isoform_174971	ACA	lightcyan	Myb_DNA-binding	Transcription
isoform_177007	AG	lightcyan	CBM49	Carbohydrate transport and metabolism
isoform_102887	GTG	lightcyan	ATR13	Translation, ribosomal structure and biogenesis
isoform_122483	AGC	lightcyan	RRM_1	General function prediction only
isoform_5055	ATG	lightcyan	PK_Tyr_Ser-Thr	General function prediction only, Transcription
isoform_121721	TCA	lightcyan	FYTT	General function prediction only
isoform_11	TGA	purple	HECT	Posttranslational modification, protein turnover, chaperones,Transcription
isoform_146908	TGA	purple	RSN1_7TM	General function prediction only
isoform_162420	ATC	purple	TCP	Transcription
isoform_167385	ATG	purple	HSP90	Posttranslational modification, protein turnover, chaperones
isoform_198965	CTT	purple	TPT	Carbohydrate transport and metabolism, Amino acid transport and metabolism
isoform_251081	CCT	purple	UPF0016	Function unknown
isoform_28540	CTC	purple	Inovirus_Gp2	Function unknown
isoform_36764	TTC	purple	Nkap_C	Function unknown
isoform_5361	TTC	purple	Reticulon	Carbohydrate transport and metabolism, Posttranslational modification, protein turnover, chaperones
isoform_108413	CCG	salmon	RMI1_N	Function unknown
isoform_99746	TTA	salmon	Fasciclin	General function prediction only, Extracellular structures
isoform_245042	GAA	salmon	RRM_1	RNA processing and modification
isoform_236555	ATG	salmon	HSP90	Posttranslational modification, protein

				turnover, chaperones
isoform_136771	TGA	tan	Glyco_transf_24	Carbohydrate transport and metabolism
isoform_219649	GGT	tan	MPLKIP	Function unknown
isoform_3833	AGA	tan	Glyco_hydro_9	Carbohydrate transport and metabolism
isoform_26815	TC	tan	SLAC1	Function unknown
isoform_7366	ATG	tan	HSP90	Posttranslational modification, protein turnover, chaperones
isoform_193867	GCA	turquoise	Auxin_resp	Signal transduction mechanisms, Transcription
isoform_16437	AGC	turquoise	RRM_1	General function prediction only
isoform_24831	TTC	turquoise	EF_TS	Translation, ribosomal structure and biogenesis
isoform_231681	CTT	turquoise	F-box	Function unknown

Table S3 Functional annotation of hubgenes related to climate factors.

Hubgene	SSR Type	Module	Pfam Domain	Functional Annotation
isoform_274508	TGC	blue	Ribosomal_S5_C	Translation, ribosomal structure and biogenesis
isoform_12373	GGTCA	blue	CBFB_NFYA	Transcription
isoform_47582	GAT	blue	EF1_GNE	Transcription
isoform_51581	ATT	blue	Snf7	Intracellular trafficking, secretion, and vesicular transport
isoform_9712	GAT	blue	Bcl-2_BAD	Posttranslational modification, protein turnover, chaperones
isoform_221678	CCA	blue	Homeobox_KN	Transcription
isoform_248044	CGG	blue	LAGLIDADG_2	General function prediction only
isoform_110445	TGA	blue	NAP	Replication, recombination and repair
isoform_17607	GAA	blue	Na_Ca_ex	Inorganic ion transport and metabolism
isoform_162345	CCT	green	UPF0016	Function unknown
isoform_219376	TGA	green	PTPlike_phytase	Posttranslational modification, protein turnover, chaperones
isoform_7662	TTAATA	green	Pkinase	Signal transduction mechanisms
isoform_965	TCA	green	NYN	Function unknown
isoform_122092	CTC	green	FH2	Signal transduction mechanisms, Cytoskeleton
isoform_102491	GGT	green	DEAD	RNA processing and modification
isoform_103046	TTC	green	EF_TS	Translation, ribosomal structure and biogenesis
isoform_272480	AGC	green	CBS	Energy production and conversion
isoform_32586	CTA	green	DUF3082	Function unknown
isoform_195328	CAG	green	HhH-GPD	Replication, recombination and repair
isoform_174971	ACA	lightcyan	Myb_DNA-binding	Transcription
isoform_177007	AG	lightcyan	CBM49	Carbohydrate transport and metabolism
isoform_102887	GTG	lightcyan	ATR13	Translation, ribosomal structure and biogenesis
isoform_122483	AGC	lightcyan	RRM_1	General function prediction only
isoform_5055	ATG	lightcyan	PK_Tyr_Ser-Thr	General function prediction only,Transcription
isoform_121721	TCA	lightcyan	FYTT	General function prediction only

Table S4. Characteristics of 16 validated genic-SSRs for *Caragana.*

Primer ID	Repeat Motif	Primer Sequence	N_A	H_O	H_E	*PIC*
CPSSR_566	AGC	F：TAACCATGCGGGGCTTTCTT<br>R：CCCACTCTCAGCAGCATCAA	3	0.381	0.292	0.501
CPSSR_1710	CTC	F：GGTCTTCACCATCCGACCAC<br>R：GCGGAGGAAGAAGCTGAAGA	4	0.256	0.278	0.603
CPSSR_2702	GGT	F：GGCATGTGGCTAGACAGTGT<br>R：CATGTCCACGACCTCCACAA	5	0.290	0.305	0.686
CPSSR_3379	TGC	F：TGACGATTCCAAAGGCCACA<br>R：ACGAGGATGCACAACTACCTG	6	0.108	0.144	0.654
CPSSR_1221	CAC	F：TACATGAGGCCACCACAACC<br>R：GGAGGGGAAGGAGTCTGGTA	4	0.371	0.283	0.438
CPSSR_2805	GTTTT	F：ACCCCATTTTACACTGCTTGC<br>R: AGGGTTAAAGAAGAGTCTGTGCA	5	0.232	0.438	0.637
CPSSR_1394	CAT	F：TGAAGAATGCACAGCTATGGC<br>R：ACACACATTCCTTGAGGGTGT	3	0.360	0.269	0.605
CPSSR_2888	TAAA	F：GCCTCGTCTTGACTCTGGTT<br>R：TCCTTCTGCCACAACCCAAG	5	0.323	0.316	0.722
CPSSR_292	ACC	F：CATGTTCCACCACCCTCCAC<br>R：CTGCTGAGGACCGGGATAAC	2	0.294	0.253	0.563
CPSSR_2728	GT	F：GGATGCGCAACAATTCCCAT<br>R：CGAAAACCCCAGCCAGATCT	11	0.278	0.560	0.863
CPSSR_3518	TGGAAA	F：GGGCTAGTGAGATTGGTGCT<br>R：GCAGAGGAATTTCCCACTGGA	4	0.198	0.170	0.682
CPSSR_3621	TTC	F：TCTTCCCCTTCATACCCTCCA<br>R：GAGCTAGTTGAGGTGGCACA	9	0.511	0.502	0.785
CPSSR_2641	GGA	F：TCGGACGAGGACTATGTGGT<br>R：TCTGCTGACCACAAATGCCA	3	0.212	0.223	0.307
CPSSR_3645	TTG	F：CTACTGGCAAGCACCATCGA<br>R：CCATCATTCTTCTGGCCCTGA	2	0.067	0.100	0.329
CPSSR_2779	GTG	F：TCCACTGGTCTCAACGTTCG<br>R：TGTGCCTCAACTGAGCACTC	3	0.347	0.306	0.518
CPSSR_1512	CCG	F：CCACCCAAATCCCCTCAACA<br>R：AGAGTGAAGAGGGGTTCGGT	3	0.056	0.086	0.279

Note: N_A, H_O, H_E and *PIC* represent allele number, observed heterozygosity, expected heterozygosity and polymorphism information content, respectively.
